# Aging Images as a Motivational Trigger for Smoking Cessation in Young Women

**DOI:** 10.3390/ijerph7093499

**Published:** 2010-09-27

**Authors:** Carine Weiss, Dirk Hanebuth, Paola Coda, Julia Dratva, Margit Heintz, Elisabeth Zemp Stutz

**Affiliations:** 1 Unit Gender and Health, Department of Epidemiology and Public Health, Swiss Tropical and Public Health Institute, Socinstr. 57, 4051 Basel, Switzerland; E-Mails: carine.weiss@unibas.ch (C.W.); dirk.hanebuth@unibas.ch (D.H.); paoletta.codina@gmail.com (P.C.); julia.dratva@unibas.ch (J.D.); 2 University of Basel, Petersplatz 1, CH-4003 Basel, Switzerland; 3 Lung Association of Basel, Kanonengasse 33, 4410 Liestal, Switzerland; E-Mail: margit.heintz@llbb.ch

**Keywords:** smoking cessation, adolescent women, aging images, recruitment, motivational stages

## Abstract

Recruiting adolescents into smoking cessation programs has been challenging, and there is a lack of effective smoking cessation interventions for this age group. We aimed to assess whether the approach of using aging images can be used to recruit young, female smokers for a smoking cessation course. In this study, 853 14- to 18-year-old subjects were photographed (2006–2007). After software-aided aging, the images evoked strong emotions, especially in subjects with an advanced motivational stage to quit. Twenty-four percent of current smokers reported that the aging images increased their motivation to quit smoking (pre-contemplation: 8%; contemplation: 32%; and preparation: 71%). In multivariate analyses, the aged images had a high motivational impact to quit smoking that was associated with an increased readiness to stop smoking and the individual’s assessment of the aging images as shocking, but not with the number of previous attempts to quit and the assessment of the pictures as realistic. However, it was not possible to recruit the study population for a smoking cessation course. We concluded that aging images are a promising intervention for reaching young women and increasing their motivation to stop smoking. However, smoking cessation courses may not be appropriate for this age group: none of the recruits agreed to take a cessation course.

## 1. Introduction

Despite continuous tobacco prevention efforts and virtually universal awareness of tobacco-related health consequences, the smoking prevalence among young people, especially women, remains high in many countries [1]. However, there are a variety of smoking prevention and cessation programs that target young people [2–5]. Various studies have shown that recruiting young people and convincing them to participate in smoking cessation programs is a demanding process [3,6,7]. While there is a demand for smoking cessation programs, there is minimal evidence of effective interventions for adolescent smokers [8–11], and this lack of evidence is likely due to the challenges in recruiting and retaining young people in cessation programs.

Although the low interest in participating in smoking cessation programs has been reported [12], little attention has been paid to the development of effective recruitment methods for adolescents [7]. In fact, most adolescents who successfully quit smoking report that they quit on their own or with the help of a friend [13]. The identification of stages and change processes related to smoking cessation based on the transtheoretical model [14,15] has been regarded as an important step for recruiting young people into smoking cessation programs [16]. To date, though, there is little evidence that tailoring messages to the different stages of change will enhance recruitment rates [17].

Gender-related factors may also be relevant. Patterns of cigarette consumption differ between men and women, *i.e.*, there is an increased prevalence of smoking in women compared to men. Young female smokers seem to be more concerned about physical appearance issues (e.g., skin aging and body weight) than their male peers [18–20]. For example, female adolescents tend to smoke to regulate their appetite and also their body weight [21], which was one reason young girls cite for not wanting to or having difficulties with quitting smoking [20,22,23]. In a focus group discussion, concerns of skin aging (wrinkles) as a consequence of smoking were reported by males and females between the ages of 17 and 24 [20]. Younger female smokers believed that wrinkles are not yet a realistic threat, but they would consider quitting smoking if their skin showed signs of wrinkles. For nonsmokers (especially women), skin aging was a reason not to take up smoking.

The present study presents the results of an evaluation of a female-specific smoking prevention and cessation project named “Smokeeffects”. This project was developed and implemented by the Lung Association of Basel, Switzerland. It aims to raise young women’s awareness of the increased risk of premature skin aging in smokers, thereby countering advertising images that depict smoking as glamorous and attractive. The concept of the project is based on the findings of the Task Force for Tobacco-Free Women and Girls [18], which focused on beauty rather than on health aspects. The approach was developed in problem-oriented workshops with peer group members and middle and high school students in 1998. As a consensus, the students regarded the association between skin aging and smoking as an adequate and effective measure to prevent young people from starting to smoke or to quit smoking. Subsequently, the application of an “aging software” was developed. The software simulates skin aging from adolescence to adulthood, both as a smoker and a nonsmoker. For the aging procedure we used the “APRIL® Age Progression Software” in the version 2.3. In 2006, this was the only software with a *smoking-related* skin aging feature.

The aim of this study was to assess the impact of showing aging images to young women in public and school events. The objective was first to sensitize female nonsmokers and smokers to the impact of smoking on their physical appearance. Then, we assessed whether this type of intervention was successful in encouraging the study population to join a smoking cessation program.

## 2. Methods

### 2.1. Study Area

**Figure 1.**
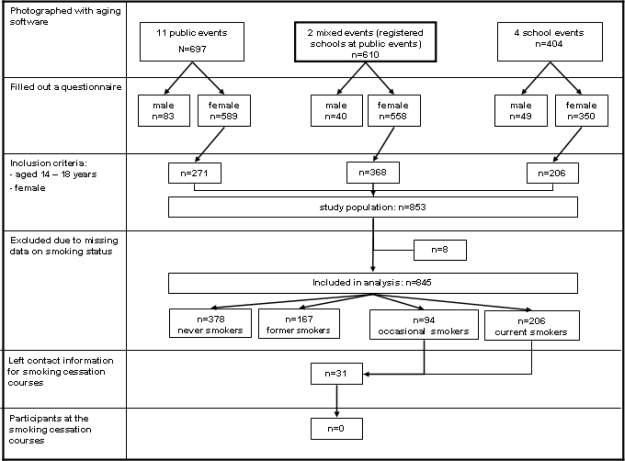
Study profile.

The intervention was conducted in the German part of Switzerland between January 2006 and December 2007 at 11 public events, four school events, and two mixed events (the school classes had registered to participate at public events) (Figure 1).

The school events took place at high schools and vocational schools; the public events included fairs, tobacco-related events (e.g., World No Tobacco Day) and one party.

### 2.2. Study Population

In total, 1,711 subjects underwent the software aging process and reported their emotional reactions and whether they intend to quit (Figure 1). The inclusion criteria for the study population (female and 14- to 18-years of age) were met by 853 subjects. Eight subjects were excluded due to missing data on their smoking status. Thus, the final study population consisted of 845 participants.

### 2.3. Aging Procedure

To illustrate how smoking induces skin changes, such as pronounced wrinkling, we worked with a current digital photograph of the participant’s face. The software generated images of how the participant would look after approximately 30 years as a smoker and as a nonsmoker, respectively. The aging process was monitored and displayed on a computer screen. If participants consented, the aging images were shown on a large screen for additional public impact. Each participant received a printed copy of the smoking and nonsmoking images. Professionals experienced in health promotion and smoking prevention that were specifically trained for this program were available to inform participants of the impact of smoking on beauty and health, as well as to give advice on smoking cessation.

### 2.4. Data Collection

The strategy of the Smokeeffects project was to evaluate the effects of aging images in adolescents. Data encompass emotional reactions on the aging images, motivational aspects concerning intention to quit (before and after the aging procedure), and manifest variables such as the registration for subsequent smoking cessation courses.

Participants were asked to complete a two-part questionnaire. The first part was administered before the aging process in order to assess the participants’ sociodemographic and educational status, smoking behaviors, intentions to quit, and opinions about the impact of smoking on beauty.

*Smoking behavior* was divided into four categories: (1) never smokers (“I have never smoked”), (2) former smokers (“I smoked occasionally, but not anymore” and “I smoked frequently, but not anymore”), (3) occasional smoker (“I smoke occasionally, less than one cigarette a day”) and (4) current smoker (“I smoke regularly”). This categorical scale had been tested in a pretest with adolescents in respect of comprehensibility and completeness [16]. *Previous attempts to quit* were categorized as the following: never tried to quit, tried once, twice or more. Based on the well-validated Prochaska Scale [14,15], the *stages of change* were assessed by asking participants whether they were considering quitting in the next 4 weeks (preparers), 6 months (contemplators), or were not considering quitting (pre-contemplators). Participants were also asked whether smoking makes them attractive and if they think that smoking can ruin their beauty.

The second part of the questionnaire was administered after the aging procedure. Participants were asked whether they perceived the aging images as realistic, whether they found the images shocking, and whether the images motivated them to quit smoking. The answers to these questions were registered on a four-point Likert-type scale (1 = “Very”, 2 = “A little” 3 = “Not at all”, and 4 = “I don’t know”).

Finally, the participants were asked whether they were interested in receiving information on an upcoming smoking cessation course. Those who provided their contact details received invitations to informative sessions evenings two weeks prior to the planned smoking cessation courses.

### 2.5. Statistical Analyses

Descriptive statistics were used to report sample characteristics for all study participants and within smoking status subgroups. Past quit attempts, opinions of the effects of smoking on beauty and attractiveness, perceptions of the aging images and the impact on motivation to quit smoking were analyzed based on the motivational stages of change. Occasional smokers were excluded from the analysis, because there was a high proportion of missing data concerning their smoking behavior (e.g., 31% of the data were missing from the question about motivational stages of change). A Chi-squared test was conducted between the missing and nonmissing data to determine whether the missing data had a potentially confounding influence. None of the tests revealed significant differences (results not presented). Statistical significance was tested using Chi-squared tests and the Kruskal-Wallis equality of population rank tests. Trends across the stages of change categories were evaluated using a nonparametric test for trends across ordered groups [24]. The “I don’t know” category was treated as missing data in order to obtain clearer ordinal scales for the variables “I find the images realistic/shocking” and “the aging images motivated me to quit smoking”. A Chi-squared trend test was conducted for the two variables concerning opinions on the effect of smoking on beauty and attractiveness. For smokers, a logistic regression analysis was performed to assess the predictors of the “high motivational impact of aging images”. The predictors were entered simultaneously. The outcome was defined as an affirmative answer to the question “aging images highly motivated me to quit smoking”. The item was chosen to reflect an increase in the motivation to quit smoking according to the concept of motivational stages of change [14,15]. Due to the small sample sizes in these categories, participants in the contemplation and preparation stages of change were grouped into a single category (intending to quit) *versus* pre-contemplators (not intending to quit). The data were analyzed using STATA software (version 9.2).

## 3. Results

### 3.1. Characteristics of the Study Population

The characteristics of the study population according to smoking status are displayed in Table 1. Overall, 44% of the participants had never smoked, 24% were currently smoking and 11% were occasional smokers. A further 18.6% reported having stopped smoking. The mean age of this sample was 16.3 (SD = 1.3), with current smokers being slightly older than the other groups. Current smokers reported smoking an average of 11.6 (SD = 6.8) cigarettes per day.

A high proportion (81%) affirmed that the issue of “smoking and beauty” mattered to them. Overall, 91% shared the opinion that smoking “ruins beauty,” and 97% reported that smoking does not make them attractive. The proportion of these opinions differed between “never-smokers” and current smokers: compared to never-smokers, more current smokers were convinced that smoking makes them attractive (23.3% *vs.* 1%), and fewer smokers thought that it would ruin their beauty (73% *vs.* 91%). The same pattern was seen regarding the participants’ perceptions of the aging images. More never-smokers reported that the images were highly realistic and shocking than the occasional and current smokers. Overall, though 49% of the participants perceived the aging images as “highly shocking”, 52% perceived the pictures as “little realistic”.

Only smokers were asked about previous attempts to quit and their intentions to quit smoking (Table 2). More than 75% of current smokers reported at least one quit attempt, compared to 40% of occasional smokers. Before the aging procedure, 46% of the current smokers reported being in the pre-contemplation stage, 41% in the contemplation stage and 8% in the preparation stage of change. Of the occasional smokers, more than a third gave no information on motivational stages, which limited the comparison of occasional smokers to current smokers.

### 3.2. Motivational Stages of Change

Table 3 shows the number of previous quit attempts for current smokers, their opinions on the effect of smoking on beauty and attractiveness, and their perception of the aging images as realistic and/or shocking according to the three motivational stages of change. However, here was only a small number of smoking women in the preparation phase (n = 17), which limited interpretation of the data somewhat. Nevertheless, significant differences occurred between subjects in the pre-contemplation, contemplation and preparation stages of change for all items except the opinion on whether smoking enhances attractiveness. There was also a significant trend for most variables across the motivational stages. There was an increasing trend in the number of previous quit attempts, an increasing trend in the opinion that smoking diminishes beauty, but no trend in the opinion that smoking makes someone attractive, and no trend in the assessment of the pictures as realistic.

The majority of the subjects in the preparation stage reported more than two quit attempts (52%). Further, all women in the preparation stage of change agreed that smoking ruins their beauty. Fewer subjects in the pre-contemplation stage perceived the aging images as shocking (61.1%) than subjects in the contemplation (78.6%) and preparation stages (82.4%). The aged images were mostly rated as a little realistic by all groups.

The aging images highly motivated more women in the preparation (70.6%) and contemplation stages (32.1%) to quit smoking compared to women in the pre-contemplation stage of change (8%). The results showed that 34.7% of pre-contemplators indicated that the aging images did not motivate them to quit smoking at all (*versus* 3.6% of women in the contemplation stage and 0% in the preparation phase). Remarkably, there was a relatively high percentage of missing data for the items regarding the perception of the aging images.

### 3.3. Impact of Aging Images on Motivation to Quit Smoking

In the multivariate analysis, we investigated predictors of the motivational impact of the aging images for quitting smoking (Table 4) in current smokers. Because there was only a small number of current smokers in the preparation stage of change (n = 17), we combined the contemplation and preparation stage into the category “intends to quit smoking”.

Those who reported an intention to quit smoking, who reported previous quit attempts and who perceived the aging images as highly realistic or highly shocking were more likely to report that “aging images highly motivated me to quit smoking”. No trend for the motivational impact to quit was seen based on the numbers of previous quit attempts. Participants who perceived the images as highly shocking were approximately three times more likely to report that the images highly motivated them to quit smoking compared to those who considered the pictures not shocking. Current smokers who indicated that smoking does not ruin beauty were much less likely to report a high motivational impact of the aging images (OR 0.07 [0.01; 0.52]).

### 3.4. Recruitment for Smoking Cessation Courses

At one public event and at one school event, 22 young women (three occasional and 19 current smokers) and nine current smokers, respectively, indicated an interest in receiving information on upcoming smoking cessation courses. However, when asked to fill out an application, only one (an occasional smoker) did so. Ultimately, neither of the two planned smoking cessation courses was conducted, because no participants attended the information sessions.

## 4. Discussion

The purpose of this study was to investigate whether the “Smokeeffects” intervention, which uses aging images, had an impact on women between 14 and 18 years of age and whether the images motivated them to quit smoking or to attend a smoking cessation course. About 85% of the young women reported that in their opinion, smoking is not attractive and ruins beauty. This trend was less pronounced in current smokers. Never smokers and former smokers found the aging images highly shocking, but half of the participants characterized the images as unrealistic. Nevertheless, aging images increased the motivation of current smokers to quit smoking, especially those in advanced motivational stages (*i.e.*, the preparation stage of change). However, no participants were recruited for a smoking cessation course.

Aging images, as proposed by Hysert [18], seem to be a promising approach for reaching a large number of young women at public events and in school events to inform them of the effects of smoking on physical appearance. Because the vast majority of the young women reported that the subject “smoking and beauty” matters to them, focusing on beauty rather than health seems to be a promising approach to get young women to quit smoking. A focus group discussion with 17- to 24-year-old men and women conducted in England showed that both smokers and nonsmokers feared skin aging. However, due to their young age, the smokers had not experienced skin aging yet, so it was a realistic but minor threat to them [20]. In our study, a high percentage of participants (especially among those in the preparation stage of change) agreed that smoking is harmful to beauty. Never-smokers had even more pronounced opinions on the negative effects of smoking on beauty compared to current smokers (91% *vs.* 73% of current smokers). They also differed on the opinion that smoking has a positive effect on attractiveness (affirmed by only 1% of never smokers *vs.* by 23% of current smokers).

The impact of aging images on the motivation to quit was seen in both the univariate and multivariate analyses. In the multivariate analysis, the increase in the motivation to quit as a result of the aging images was associated with the intention to quit in the next 6 months, numbers of previous quit attempts and the perception of the aging images as shocking and as realistic. Even among those in the pre-contemplation stage, 50% of subjects reported that they were (minimally) slightly more motivated to quit after their confrontation with the aging images. Although these women did not want to consider stopping smoking in the near future, it appears that this approach was able to evoke strong emotions. These results highlight the potential of aging images not only to reach young women and to evoke strong emotions but also to prompt stage transition.

The higher motivation of subjects in the preparation stage of to stop smoking change has been previously shown and is consistent with the underlying theory of the stages behind behavioral change [14,16]. Our findings confirm this paradigm for young, smoking women. In addition, they point to the phenomenon that not only behavior and opinions but even the perception of (aging) images as realistic or shocking is modified by the stage of change. In the trend analysis, pre-contemplators revealed their reluctance or resistance to accept that smoking might have an impact on beauty. Thus, it seems that they are less likely to be influenced by aging images. These findings underscore the importance of tailoring approaches for subjects of different stages of change, as emphasized by Dijkstra *et al.* [25,26].

The proportion of pre-contemplators (48%) was similar to a previous study conducted in the US [16]. Developing the intention to quit is one of the first identifiable steps in the cessation process [27]. Using aging images for young women likely affects this early step, *i.e.*, targeting young women’s perceptions regarding the effects of smoking thereby settles the first steps in the cessation process. Because no follow-up data are available, the impact of this intervention on later cessation outcomes and changes in the motivational stage of change could not be assessed. A further limitation of this study was the small sample size of preparers, which may have affected the power of the analysis.

The impact of the aging images may also depend on the type of event where they were presented. In school events, their impact may be less pronounced, because attendance is less voluntary. In our study, the proportion of current smokers was similar for each type of event (about a third). When including the type of event in the multivariate analysis, the odds ratio for school events was 1.45 (CI 0.61–3.43), thereby indicating a lower impact of the aging images for school events.

Similar to findings in other studies [13,28], the majority of smokers had made at least one previous attempt to quit but relapsed. Only a very small proportion of former smokers reported a successful cessation (3% of regular and 16% of occasional smokers). Adolescents’ negative attitudes towards smoking cessation programs have been documented [12]. It appears that young people feel confident that they can stop smoking without assistance. However, the small proportion of those who actually quit (18.4%) indicates that adolescents underestimate the difficulty of quitting [28].

Despite of the success of reaching young women and motivating them to quit smoking, it is crucial to answer why the facilitators failed to recruit young women for a cessation course. Although direct face-to-face contact has been mentioned in the literature as a promising intervention [5], it seems that during the events, sufficient weight was not given to the recruitment process. During the events, visitors encountered many other “eye-catching” items, and the professionals’ limited time precluded longer individual counseling. Given that further efforts are needed to target those in the advanced motivational stage, our findings underscore the importance of assessing the stages of change at the beginning of the program and of adapting communicational, as well as organizational, skills according to these stages. These assessments have also been suggested by other researchers [16,25,26,29].

To learn why the study participants did not attend the cessation courses and how strongly they consider their need to quit, a survey was conducted at one of the schools, specifically, a school of health technology. The results of this survey showed that negative attitudes towards smoking cessation programs are widespread. Young female smokers and nonsmokers alike reported low interest in group courses, whereas quitting in pairs or with the help of a friend was more popular. Two-thirds of the survey participants did not answer the question on how a suitable smoking cessation course should be designed for them. This may suggest that young women generally devote little thought to the idea of cessation courses, or they may even be unaware that such programs exist. Smoking cessation courses do not seem to be appealing for adolescents. As such, they may not be an appropriate method for recruiting them.

In conclusion, this study suggests that aging images are a promising approach for reaching out and informing young women of the dangers of smoking. The photos also enhanced their motivation to quit smoking. Second, more attention should be given to motivational stages of change that already exist in the recruitment process for smoking cessation interventions, such that information can be tailored to the reported stage of change. Finally, the methods for smoking cessation should not only consist of courses; other methodologies that are better suited to adolescents must be developed as well.

## Figures and Tables

**Table 1.** Characteristics of the study population organized by smoking status.

		**Never smokers n = 378**	Former smokers* n = 167	**Occasional smokers n = 94**	**Current smokers n = 206**	**Total n = 845**	**p-value^§^**
Age M (SD)		16 (1.4)	16 (1.3)	16.5 (1.0)	16.7 (1.1)	16.3 (1.3)	

		n (%)	n (%)	n (%)	n (%)	n (%)	

Education/occupation N (%)	In school	267 (70.6)	122 (73.1)	50 (53.2)	107 (51.9)	546 (64.6)	P < 0.001
	In training/working part-time	102 (27)	38 (22.8)	38 (40.4)	83 (40.3)	261 (30.9)	
	Working full-time	3 (0.8)	5 (3)	5 (5.3)	9 (4.4)	22 (2.6)	
	Housewife	0	0	0	0	0	
	Unemployed	6 (1.6)	0	1 (1)	5 (2.4)	12 81.4)	
	*Missing*	*0*	*2 (1.2)*	*0*	*2 (1)*	*4 (0.5)*	

In my opinion, smoking makes me attractive.	Yes	3 (0.8)	15 (9)	13 (13.8)	48 (23.3)	79 (9.4)	p < 0.001
	No	366 (96.8)	145 (86.8)	73 (77.7)	145 (70.4)	729 (86.3)	
	*Missing*	*9 (2.4)*	*7 (4.2)*	*8 (8.5)*	*13 (6.3)*	*37 (4.4)*	

In my opinion, smoking ruins beauty.	Yes	344 (91)	147 (88)	78 (83)	151 (73.3)	720 (85.2)	p < 0.001
	No	29 (7.7)	17 (10.2)	15 (16)	45 (21.8)	106 (12.5)	
	*Missing*	*5 (1.3)*	*3 (1.8)*	*1 (1)*	*10 (4.9)*	*19 (2.3)*	

The subject “smoking and beauty” is important.	Yes	305 (80.7)	140 (83.8)	76 (80.9)	159 (77.2)	680 (80.5)	n.s.
	No	47 (12.4)	17 (10.2)	10 (10.6)	29 (14.1)	103 (12.2)	
	*Missing*	*26 (6.9)*	*10 (6)*	*8 (8.5)*	*18 (8.7)*	*62 (7.3)*	

I found the aging images realistic.	Highly	114 (30.2)	43 (25.8)	21 (22.3)	47 (22.8)	225 (26.6)	p < 0.001
	A little	189 (50)	80 (47.9)	57 (60.6)	85 (41.3)	411 (48.6)	
	Not at all	21 (5.6)	6 (3.6)	7 (7.5)	28 (13.6)	62 (7.3)	
	I don’t know	16 (4.2)	7 (4.2)	4 (4.3)	11 (5.3)	38 (4.5)	
	*Missing*	*38 (10.1)*	*31 (18.6)*	*5 (5.3)*	*35 (17)*	*109 (12.9)*	

I found the aging images shocking.	Highly	196 (51.9)	89 (53.3)	42 (44.7)	84 (40.8)	411 (48.6)	n.s.
	A little	106 (28)	45 (27)	30 (31.9)	63 (30.6)	244 (28.9)	
	Not at all	23 (6.1)	7 (4.2)	6 (6.4)	22 (10.7)	58 (6.9)	
	I don’t know	14 (3.7)	5 (3)	1 (1.1)	7 (3.4)	27 (3.2)	
	*Missing*	*39 (10.3)*	*21 (12.6)*	*15 (16)*	*30 (14.6)*	*105 (12.4)*	

* Former smokers includes the category “I smoked occasionally, but not anymore” and “I smoked regularly, but not anymore.” Some totals are not 100% due to rounding.

§ Chi^2^ test.

**Table 2.** Quit attempts and motivational stages of change before the aging procedure and organized by smoking status.

		**Occasional smokers n = 94 (31%)**	**Current smokers n = 206 (69%)**	**Total n = 300**
Number of previous attempts to quit	None	38 (40.4)	49 (23.8)	87 (29)
	One	19 (20.2)	79 (38.3)	98 (32.7)
	Two	4 (4.3)	31 (15.1)	35 (11.7)
	More than two	11 (11.7)	46 (22.3)	57 (19)
	*Missing*	*22 (23.4)*	*1 (0.5)*	23 (7.7)

Motivational stages of change	Pre-contemplation	27 (28.7)	95 (46.1)	122 (40.7)
	Contemplation	25 (26.6)	84 (40.8)	109 (36.3)
	Preparation	8 (8.5)	17 (8.3)	25 (8.3)
	*Missing*	*34 (36.2)*	*10 (4.9)*	*44 (14.7)*

**Table 3.** Previous quit attempts, opinion about smoking and beauty, and the perception of aging images organized by motivational stages of change in current smokers (N = 196)^a^.

		**Pre-contemplation n = 95**	**Contemplation n = 84**	**Preparation n = 17**	**p value**	**p value for trend**
Mean age		16.7 (1)	16.8 (1.2)	16.7 (1)		

		n (%)	n (%)	n (%)		

Number of previous attempts to quit	None	33 (34.7)	13 (15.5)	2 (11.8)	p < 0.001**†**	p < 0.001*
	One	36 (37.9)	35 (41.7)	4 (23.5)		
	Two	12 (12.6)	14 (16.7)	2 (11.8)		
	More than two	13 (13.7)	22 (26.2)	9 (52.9)		
	*Missing*	*1 (1)*	*0*	*0*		

In my opinion, smoking makes me attractive	Yes	22 (23.2)	21 (25)	2 (11.8)	n.s.**^§^**	n.s.**‡**
	No	68 (71.6)	59 (70.2)	13 (76.5)		
	*Missing*	*5 (5.3)*	*4 (4.8)*	*2 (11.8)*		

In my opinion, smoking ruins beauty	Yes	59 (62.1)	67 (79.8)	17 (100)	n.s.**^§^**	p < 0.001**‡**
	No	30 (31.6)	15 (17.9)	0		
	*Missing*	*6 (6.3)*	*2 (2.4)*	*0*		

I found the aging images realistic	Highly	18 (19)	21 (25)	3 (17.7)	n.s.**†**	n.s.*, b
	A little	38 (40)	33 (39.3)	10 (58.8)		
	Not at all	17 (17.9)	9 (10.7)	1 (5.9)		
	I don’t know	7 (7.4)	3 (3.6)	1 (5.9)		
	*Missing*	*15 (15.8)*	*18 (21.4)*	*2 (11.8)*		

I found the aging images shocking	Highly	24 (25.3)	42 (50)	12 (70.6)	p < 0.01**†**	p < 0.001*, b
	A little	34 (35.8)	24 (28.6)	2 (11.8)		
	Not at all	15 (15.8)	6 (7.1)	1 (5.9)		
	I don’t know	6 (6.3)	1 (1.29	0		
	*Missing*	*16 (16.8)*	*11 (13.1)*	*2 (11.8)*		

Aging images motivated me to quit smoking	Highly	8 (19)	27 (32.1)	12 (70.6)	p < 0.001**†**	p < 0.001*, b
	A little	29 (30.5)	32 (38.1)	5 (29.4)		
	Not at all	33 (34.7)	3 (3.6)	0		
	I don’t know	9 (9.5)	7 (8.3)	0		
	*Missing*	*16 (16.8)*	*15 (17.9)*	*0*		

a Eighteen participants were excluded due to missing data on the motivational stages of change.

b The “I don’t know” category was treated as missing data.

§ Chi^2^ test.

† Kruskal Wallis rank test (p-value with ties).

* Nptrend (nonparametric test for trend across ordered groups).

‡ Chi^2^ test of trend (tabodds).

**Table 4.** Factors predicting a high motivational impact of aging images on quitting smoking in current smokers (N = 147**§**).

**Predictors**		**Adjusted odds ratio† (95% confidence interval).**
Smoking cessation intention**a, b**	Does not intend to quit smoking	1
	Intends to quit smoking	7.91 [2.53, 24.80]***

Opinion on the effect of smoking on beauty and attractiveness	Smoking ruins beauty	1
	Smoking does not ruin beauty	0.07 [0.01, 0.52]**
	Smoking does not make me attractive	1
	Smoking makes me attractive	0.82 [0.08, 8.16]

Number of previous quit attempts	None	1
	One	0.80 [0.19, 3.43]
	Two	0.38 [0.07, 2.11]
	More than two	3.43 [0.78, 15.13]

Perception of aging images**c**	Aging images are not highly realistic	1
	Aging images are highly realistic	3.24 [1.07, 9.76]***
	Aging images are not highly shocking	1
	Aging images are highly shocking	3.48 [1.27, 9.59]***

* p < 0.05,

** p < 0.01, and

*** p < 0.001.

§ Logistic Regression includes N = 147 observations in with complete data for all variables.

† Odds ratio adjusted for age and education/occupation.

a The outcome is an affirmative answer to the question “aging images highly motivated me to quit smoking.”

b Due to small numbers, participants from the contemplation and preparation stage of change were put into one category (intending to quit) *vs.* pre-contemplators (not intending to quit).

c Includes the categories “a little”, “not at all”, and “I don’t know.”
